# Electrophysiological evaluation of the auditory pathway in newborns and infants with peri-intraventricular hemorrhage and/or periventricular leukomalacia

**DOI:** 10.1016/j.clinsp.2025.100853

**Published:** 2025-12-18

**Authors:** Rosanna Giaffredo Angrisani, Natalia Olival Balzarini, Carla Regina Tragante, Valdenise Martins Laurindo Tuma Calil, Werther Brunow de Carvalho, Carla Gentile Matas

**Affiliations:** a Departamento de Fisioterapia, Fonoaudiologia e Terapia Ocupacional da Faculdade de Medicina da Universidade de São Paulo, São Paulo, SP, Brazil; b Departamento de Pediatria da Faculdade de Medicina da Universidade de São Paulo, São Paulo, SP, Brazil

**Keywords:** Evoked potentials, Auditory, Newborns, Intracranial hemorrhage, Leukomalacia, Periventricular, Hearing

## Abstract

**Objective:**

To evaluate and monitor, through electrophysiological assessment of hearing, the integrity of the peripheral and central auditory pathways in infants with Peri-intraventricular hemorrhage and/or Periventricular Leukomalacia (PIVH/PVL) who stayed in a Neonatal Intensive Care Unit (NICU), aiming to verify the occurrence of possible neural dysfunctions in this system.

**Material and methods:**

This prospective longitudinal study evaluated preterm Newborns (NBs) and infants at the time of hospital discharge and after 3- and 6-months. The Study Group (SG) had 12 females and 11 males, with gestational age between 25- and 33-weeks, and a mean gestational age of 29.82-weeks at birth. The Control Group (CG) had 26 healthy NBs, distributed in 13 females and 13 males, with gestational age between 27- and 33-weeks and a mean of 30.67-weeks of gestational age at birth. All participants underwent Auditory Brainstem Response (ABR) and Cortical Auditory Evoked Potentials (CAEP) P1, N1, P2, at the time of hospital discharge, and 3- and 6-months after discharge. Each group’s results were compared using statistical tests.

**Results:**

Evolutionary study of mean ABR and CAEP latencies in infants in the study and control group showed a similar pattern over the six months after hospital discharge.

**Conclusion:**

The comparison of brainstem and cortical potentials showed that auditory function is symmetrical in the peripheral and central portions of the auditory pathway in both groups. The maturation of the ABR and CAEP waves in both groups developed in a very similar way over the six months after hospital discharge.

## Highlights

• Prematurity is a risk factor for lifelong hearing damage.
• Hearing monitoring is necessary until around 3-years of age.
• Hearing monitoring after peri-intraventricular hemorrhage is essential.

## Introduction

Peri-Intraventricular Hemorrhage (PIVH) is one of the most common complications in very premature Newborns (NB) due to the immaturity and lability of the cerebral blood vessels, which are unable to provide the necessary oxygen to the brain, being frequent in approximately 13 % to 29.8 % of NBs with less than 32-weeks of Gestational Age (GA) and in 44.68 % of other premature babies^1^^,^^2^. PIVH can be classified according to the degree of severity as Grade I – limited to the area of the germinal matrix (subependymal hemorrhage); Grade II – with rupture of the ventricular cavities, without ventricular dilation; Grade III – with dilation of the ventricles; and Grade IV – reaching the brain parenchyma, causing intraparenchymal hematoma (with involvement of the periventricular white matter). The prognosis of PIVH varies according to the degree of the lesion^3^^,^^4^.

Periventricular Leukomalacia (PVL) is a disorder characterized by multifocal areas of necrosis, forming cysts in the deep cerebral white matter, and can occur in more than 25 % of extremely premature NBs^5^^,^^6^.

Both encephalopathies have a negative impact on the neurodevelopment of premature NBs, including hearing and language development, as they may affect the acoustic information pathway, causing changes in the brainstem, cortical areas, or both^5^, ^6^, ^7^, ^8^. Thus, the normal functioning of central auditory structures is of utmost importance for the child to acquire perceptual skills^9^.

PIVH/PVL is one of the causes of peripheral and central auditory changes in premature infants. It is a risk factor for changes in the central auditory nervous system, potentially impairing the development of auditory skills necessary for adequate acoustic information processing and language development^9^^,^^10^.

Therefore, it is essential to assess the integrity of the peripheral and central auditory system to monitor language development, especially in the first 2-years of life. The child must be able to pay attention, detect, discriminate, and locate sounds, and memorize and integrate auditory experiences to recognize and understand speech^9^.

The authors hypothesized that the central auditory pathway in premature infants with PIVH/PVL may be changed compared to infants without risk.

## Objectives

To evaluate and monitor, through electrophysiological assessment of hearing, the integrity of the peripheral and central auditory pathways in infants with PIVH/PVL who stayed in a Neonatal Intensive Care Unit (NICU), aiming to verify the occurrence of possible neural dysfunctions in this system.

## Material and method

This prospective longitudinal study evaluated preterm NBs and infants at the time of hospital discharge and after 3- and 6-months. Data collection procedures began only after the Research Ethics Committee of the Medical School of the University of São Paulo (CAPPesq HCFMUSP) approved the project under n° 4.408.694/2020 ‒ CAAE: 38,842,020.4.0000.0068.

Following the ethical principles for human research, the mothers and/or guardians who agreed to their NBs’ and infants’ participation in this research signed an informed consent form describing all procedures. Thus, they consented to this research and the subsequent dissemination of the results, in accordance with the Brazilian Resolution 466/12.

The sample was recruited by convenience sampling among NBs and infants from the nursery attached to the maternity ward at the Clinics Hospital of the Medical School of the University of São Paulo. They were evaluated at the time of hospital discharge and after 3- and 6-months.

Even after the end of this study, children with a suspicion of hearing impairment or changes in nerve sound conduction continued having their auditory development assessed and monitored at the audiology department of the Teaching and Research Center of the Department of Physical, Speech-Language-Hearing, and Occupational Therapy of the Medical School of the University of São Paulo (CDP-FOFITO).

The initial proposal was to monitor the hearing of infants up to 12-months old with periodic electrophysiological exams. However, several setbacks led to losing contact with children in both groups, with the COVID-19 pandemic having the greatest impact on the absence and refusal to continue participating in the research. Guardians mainly claimed fear of contagion, lack of someone to take the child to the exam, fear of losing their job (even though they would receive a medical certificate), and relevant financial issues.

Thus, the initial case series comprised a Study Group (SG) with 12 female and 11 male NBs, with gestational age between 25- and 33-weeks, and a mean gestational age of 29.82-weeks at birth, totaling 23 NBs – 19 with only PIVH and four with PIVH and PVL. Their mean corrected gestational age was 41.82-weeks at the first evaluation after hospital discharge. Eleven infants attended the second evaluation after 3-months, with a mean corrected gestational age of 55.82-weeks at the time of the examination. Six infants attended the third evaluation, 6-months after discharge, with a mean corrected gestational age of 69.32-weeks at the time of the examination.

**Fig. 1:**
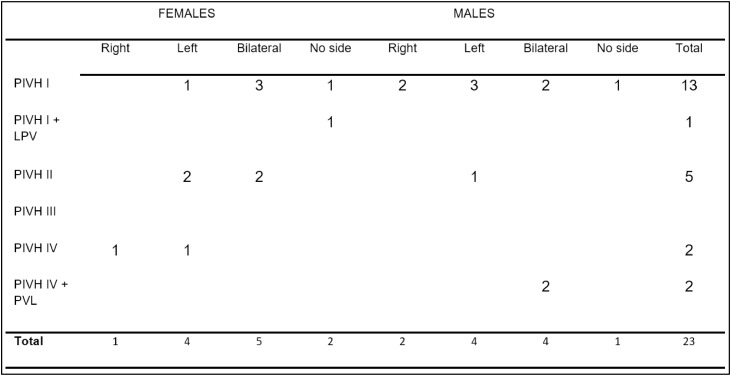
Distribution of PIVH grades per sex in the study group.

It also had a Control Group (CG) with 26 healthy NBs without PIVH/PVL in the first evaluation after hospital discharge, distributed in 13 female and 13 male NBs, with gestational age between 27- and 33-weeks and a mean of 30.67-weeks of gestational age at birth. Their mean corrected gestational age was 41.78-weeks at the first evaluation after hospital discharge. Twelve infants attended the second evaluation after 3-months, with 57.67-weeks of corrected gestational age at the time of the examination. Only five infants attended the third evaluation 6-months after discharge, with a mean of 68.75-weeks of corrected gestational age at the time of the examination. In the fourth evaluation, 12-months after hospital discharge, no patient in either group agreed to complete the study (Fig. 1).

### Procedures

The clinical/diagnostic equipment Navigator-Pro, Biologic brand, captured the Auditory Brainstem Response (ABR). All NBs were prepared for the tests by cleaning their skin with abrasive paste and attaching disposable pediatric electrodes Meditrace-200, Kendal brand, to the Frontal region (Fpz) and the right and left mastoids (M2 and M1), following the IES 10‒20 (International Electrode System) standard^11^. The impedance of the electrodes was kept below 3 kΩ. The acoustic stimuli were rarefied polarity clicks, presented monaurally through a pair of insert earphones model 3A to elicit the responses, at 80 dBnHL, at a presentation rate of 27.7 clicks/s, duration of 0.1 milliseconds (ms), high-pass filters of 100 Hz, and low-pass filters of 1500 Hz to avoid excess artifacts, using a total of 2048 stimuli. The recording window had 12 ms, and the analysis approached the absolute latencies of waves I, III, V, and interpeak intervals I‒III, III‒V, and I‒V. ABR was classified as normal and abnormal, following the criteria established by Rosa et al. (2014)^12^.

The reference electrodes were positioned on the right and/or left mastoids (M2 and M1), the active electrode was positioned on the vertex-high Forehead (Fz), and the ground electrode was positioned on the mastoid of the contralateral ear to obtain the long-latency auditory evoked potentials, P1, N1, P2. The acoustic stimuli used were syllables /ba/ at 70 dBnHL. The evaluation parameters were 300 /ba/ stimuli, in a recording window of 512 ms, presentation rate of 1.1 stimuli per second, trapezoidal stimulus envelope, band-pass filter from 1 to 30 Hz, gain of 100,000, and a response analysis window of −100 ms pre-stimulus and 500 ms post-stimulus. The lead researcher and two examiners visually verified whether Cortical Auditory Evoked Potentials (CAEP) were present and analyzed their latency. CAEP latency values were marked considering the point of maximum amplitude, determined as the difference between the baseline and the maximum positive or negative point according to the component analyzed.

### Eligibility criteria

1 – The study included all preterm NBs – children born up to 36 6/7-weeks of gestational age, according to the World Health Organization (2014)^13^.
2 – For SG, the presence of PIVH and/or PVL risk indicators. For CG, the absence of PIVH and/or PVL, craniofacial malformations, and risk indicators for retrocochlear changes, according to the Joint Committee on Infant Hearing (JCIH, 2019)^14^.
3 – Bilateral presence of transient evoked otoacoustic emissions for both groups to ensure normal cochlear function.

### Exclusion criteria

1 – All full-term NBs – children born after 37-weeks of gestational age – were excluded^13^.
2 ‒ NBs with conductive and/or cochlear changes and other risk indicators for hearing impairment were excluded from the sample, according to the criteria established by the JCIH. (2019)^14^.

All subjects underwent ABR with click stimuli and CAEP with speech stimuli (syllable /ba/). Each group’s results were compared using statistical tests.

### Statistical analysis

Quantitative data analysis was initially performed by obtaining descriptive measures (mean and standard deviation) for each group, using the ANOVA statistical test with Tukey's test when more than two intra-subject variables were analyzed, at the three evaluation moments (immediately after hospital discharge, 3rd-month after hospital discharge, and 6th-month after hospital discharge).

The probability of rejecting the null hypothesis was set at 5 %. Statistically significant values were marked with an asterisk (*), and a tendency towards significance was marked with a hash (#).

## Results

The study sample initially had 23 NBs in SG and 26 NBs in CG.

Two methodologies were used to analyze the results, the first part consisted of a cross-sectional data analysis, and the second, a longitudinal analysis.

Initially, the absolute latencies of waves I, III, V, and the interpeak intervals I‒III, III‒V, I‒V were analyzed in each group, separately for each ear, to characterize the responses of the Auditory Brainstem Response (ABR) in the study population. These analyses did not show any statistical difference between the ears in either test. Therefore, the right and left ear values were grouped for subsequent analyses, while maintaining the comparison between the Experimental Group (EG) and the Control Group (CG).

Table 1 shows that there was no significant difference in ABR parameters in the first assessment (immediately after hospital discharge – NB), except for interpeak III‒V (*p* = 0.042), with a lower latency value in the SG.

**Table 1:** Comparative study of the mean absolute latencies of ABR waves I, III, V and interpeak intervals I‒III, III‒V, I‒V between infants from the control and study groups, in the first evaluation (after hospital discharge – NB).

	SG (*n* = 46)		CG (*n* = 52)		
ABR	Mean (ms)	SD	Mean (ms)	SD	p-value
**Wave I**	1.66	0.16	1.64	0.11	0.648
**Wave III**	4.38	0.34	4.31	0.27	0.403
**Wave V**	6.50	0.18	6.60	0.13	0.586
**Interpeak interval I‒III**	2.68	0.27	2.67	0.26	0.824
**Interpeak interval III‒V**	2.14	0.28	2.37	0.44	**0.042**^a^
**Interpeak interval I‒V**	4.88	0.35	4.95	0.38	0.580

n, Number of the sample; SD, Standard Deviation.

a Statistically significant value; ANOVA test.

No differences in ABR parameters were found in the second evaluation (3-months after hospital discharge – 3 m), except for wave I (*p* = 0.012), with a lower latency value in the CG (Table 2).

**Table 2:** Comparative study of the mean absolute latencies of ABR waves I, III, V and interpeak intervals I‒III, III‒V, I‒V between infants from the control and study groups, in the second assessment (3-months after hospital discharge – 3 m).

	SG (*n* = 22)		CG (*n* = 24)		
ABR	Mean (ms)	SD	Mean (ms)	SD	p-value
**Wave I**	1.65	0.13	1.56	0.10	**0.012**^a^
**Wave III**	4.22	0.37	4.09	0.27	0.173
**Wave V**	6.25	0.39	6.21	0.26	0.665
**Interpeak interval I‒III**	2.49	0.27	2.53	0.24	0.649
**Interpeak interval III‒V**	2.02	0.17	2.22	0.46	0.050
**Interpeak interval I‒V**	4.58	0.23	4.66	0.26	0.337

n, Number of the sample; SD, Standard Deviation.

a Statistically significant value; ANOVA test.

The results of the comparison between the SG and CG described above in Table 3 show a significant difference between ABR wave V and interpeak interval III‒V in the third evaluation, with lower latency values in the SG (after 6-months of hospital discharge – 6 m).

**Table 3:** Comparative study of the mean absolute latencies of ABR waves I, III, V and interpeak intervals I‒III, III‒V, I‒V between infants from the control and study groups, in the third assessment (6-months after hospital discharge).

	SG (*n* = 12)		CG (*n* = 10)		
ABR	Mean (ms)	SD	Mean (ms)	SD	p-value
**Wave I**	1.54	0.20	1.58	0.07	0.572
**Wave III**	3.96	0.17	4.02	0.19	0.475
**Wave V**	5.90	0.17	6.11	0.09	**0.002**^a^
**Interpeak interval I‒III**	2.49	0.32	2.45	0.19	0.732
**Interpeak interval III‒V**	1.92	0.16	2.09	0.13	**0.016**^a^
**Interpeak interval I‒V**	4.44	0.30	4.54	0.11	0.302

n, number of the sample; SD, Standard Deviation.

a Statistically significant value; ANOVA test.

Table 4 below shows the minimum and maximum values of the CAEP parameters obtained in the study population, in the right and left ears.

**Table 4:** Minimum and maximum CAEP values in the control and study groups.

	RE	LE	RE	LE	RE	LE
Groups	Min–Max	Min–Max	Min–Max	Min–Max	Min–Max	Min–Max
	**P1 (ms)**	**P1 (ms)**	**N1 (ms)**	**N1 (ms)**	**P2 (ms)**	**P2 (ms)**
**CG**	60.6–237	74.2–236	96.9–274	120–302	141–395	257–412
**SG**	92.9– 217	72.7–213	142–306	103–290.6	163–305.5	257–314

ms, Milliseconds.

**Fig. 2:**
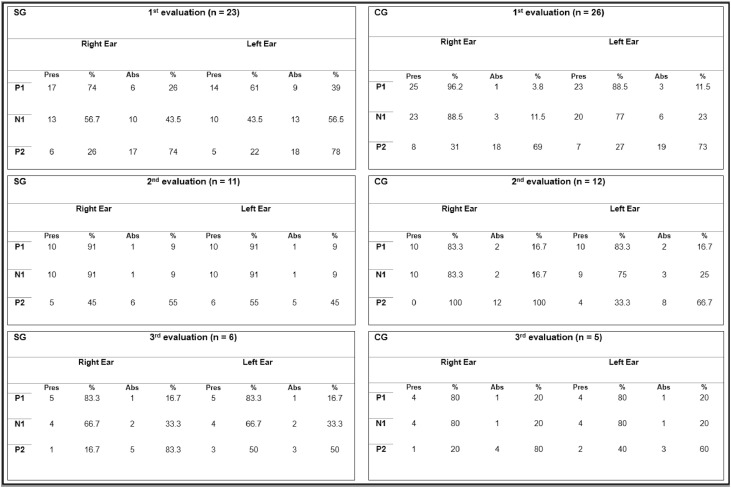
Percentage of presence and absence of CAEP components in the study and control groups in each assessment. n, Number of the sample; Pres, Presence; Abs, Absence.

Fig. 2 shows the percentage of presence and absence of CAEP components in the SG and CG in each assessment.

Note that the N2 component was not visualized in the three periods evaluated in either group.

The longitudinal data analysis evaluated how the maturation process of the ABR and CAEP parameters occurs in each group, by comparing their mean wave latency values in the first assessment after discharge (called NB), in the second assessment 3-months after discharge (called 3 m), and in the third assessment 6-months after hospital discharge (called 6 m).

The results in Table 5 show no significant decrease in the latencies of ABR wave I (*p* = 0.292) in SG throughout the study period. There was a significant decrease in the latencies of waves III and V (*p* = 0.002 and < 0.0001, respectively). Likewise, there was a significant difference in the interpeak intervals I‒III, III‒V, and I‒V (*p* = 0.006, *p* = 0.006, and < 0.0001, respectively). There was no statistically significant decrease in the CAEP P1, N1, and P2 latencies in the three study periods.

**Table 5:** Evolutionary study of the mean ABR and CAEP latencies in infants of the study group.

SG	NB (*n* = 46)		3 m (*n* = 22)		6 m (*n* = 12)		
	Mean	SD	Mean	SD	Mean	SD	p-value
Wave I	1.65	0.15	1.58	0.39	1.54	0.20	0.292
Wave III	4.36	0.37	4.21	0.37	3.96	0.17	0.002^a^
Wave V	6.55	0.46	6.22	0.38	5.90	0.17	<0.0001^a^
Interpeak interval I‒III	2.70	0.31	2.36	0.58	2.49	0.32	0.006^a^
Interpeak interval III‒V	2.17	0.28	2.01	0.17	1.97	0.13	0.006^a^
Interpeak interval I‒V	4.93	0.39	4.56	0.20	4.44	0.30	<0.0001^a^
P1	136.71	42.44	130.27	41.39	109.55	42.55	0.308
N1	202.12	54.36	205.10	67.13	154.05	48.25	0.102
P2	250.08	61.12	283.77	82.97	258.67	70.90	0.568

n, Number of the sample; SD, Standard Deviation.

a Statistically significant value (*p* ≤ 0.05); ANOVA test.

According to the Tukey test (two-by-two comparison), a significant difference occurred in the maturation of wave III between the first (NB) and third evaluations (6 m) (*p* < 0.01); a significant difference occurred in the maturation of wave V between the immediately after discharge and after 3-months (NB and 3 m) (*p* < 0.05), the same occurring between the first and third evaluations (NB and 6 m) (*p* < 0.01) and between the second and third evaluations (3 m and 6 m) (*p* < 0.05). Regarding interpeak interval I‒III, a significant difference was found only between immediately after discharge and after 3-months (NB and 3 m) (*p* < 0.05); the interpeak interval III‒V matured significantly between immediately after discharge and after 6-months (NB and 6 m) (*p* < 0.05); lastly, the differences in the interpeak interval I‒V occurred between NB and 3 m (*p* < 0.01) and NB and 6 m (*p* < 0.01), with a decrease in ABR latencies. According to the Tukey test (two-by-two comparison), no significant differences were found between the CAEP components in the three study periods.

The results in Table 6 show a significant decrease in CG in the latencies of ABR waves III and V and interpeak intervals I‒III and I‒V (*p* < 0.0001 and < 0.0001, *p* = 0.002 and *p* = 0.0001, respectively) throughout the study period. There was no statistically significant decrease in the CAEP P1, N1, and P2 latencies in the three study periods.

**Table 6:** Evolutionary study of mean ABR latencies in infants in the control group.

CG	NB (*n* = 52)		3 m (*n* = 24)		6 m (*n* = 10)		
	Mean	SD	Mean	SD	Mean	SD	p-value
**Wave I**	1.63	0.12	1.55	0.10	1.57	0.05	0.106
**Wave III**	4.31	0.25	4.09	0.27	4.02	0.19	<0.0001^a^
**Wave V**	6.58	0.36	6.20	0.26	6.12	0.10	<0.0001^a^
**Interpeak interval I‒III**	2.68	0.23	2.53	0.24	2.45	0.19	0.002^a^
**Interpeak interval III‒V**	2.31	0.37	2.23	0.48	2.09	0.13	0.200
**Interpeak interval I‒V**	4.95	0.39	4.64	0.26	4.54	0.11	0.0001^a^
**P1**	130.82	52.82	120.47	50.16	118.45	32.46	0.659
**N1**	201.5	53.16	194.30	61.93	203.15	42.44	0.878
**P2**	280.5	67.9	306.5	77.32	251.82	36.84	0.570

n, Number of the sample; SD, Standard Deviation.

a Statistically significant value (*p* ≤ 0.05); ANOVA test.

According to the Tukey test (two-by-two comparison), wave I did not have statistically significant periods, with gradual evolution throughout the study; as for wave III, a significant difference occurred in the period between the first and second evaluation (NB and 3 m) (*p* < 0.05) and between the first and third evaluation (NB and 6 m) (*p* < 0.01). As for the decrease in wave V latencies, a large difference occurred between immediately after discharge and after 3-months (NB and 3 m) (*p* < 0.01), the same occurring between the first and third evaluations (NB and 6 m) (*p* < 0.01) and between the first and third evaluations (NB and 6 m) (*p* < 0.01). Also, a significant difference in interpeak interval I‒III was only evident between immediately after discharge and after 3-months (NB and 3 m) (*p* < 0.05). Lastly, the differences in interpeak interval I‒V occurred between NB and 3 m (*p* < 0.05) and NB and 6 m (*p* < 0.01), with a decrease in ABR latencies.

**Fig. 3:**
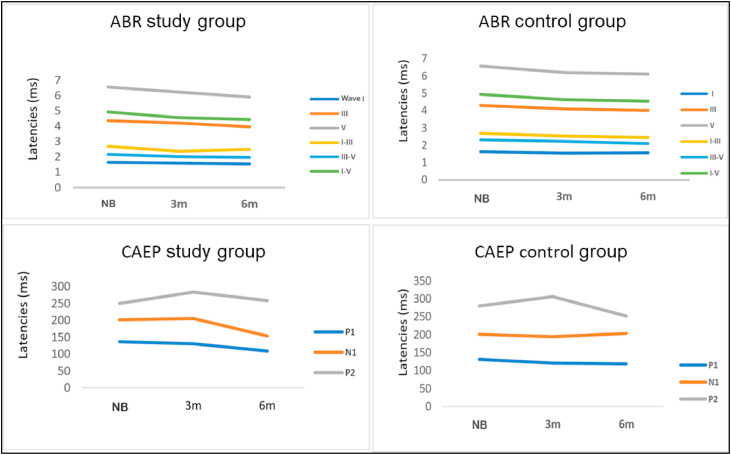
Evolutionary study of mean ABR and CAEP latencies in infants in the study and control group.

Fig. 3 illustrates the evolution of the BAEP and CAEP parameters in the Control and Study groups in the three periods analyzed.

## Discussion

Despite the setbacks that arose throughout the research, its results will hopefully contribute to advances in knowledge about the central auditory pathway functioning and neuroaudiological monitoring in infants who suffered PIVH and/or PVL due to prematurity, mitigating the impact of these problems on language development.

This study included 23 infants, making up the Study Group. PIVH affected 35 % of them on the left side and 13 % on the right side; bilateral PIVH occurred in 39 %, and in 13 % there was no defined side. These findings are noteworthy when considering that the left side of the cerebral cortex is responsible for language, having been affected in the vast majority of the infants evaluated. There may be losses along the auditory pathway even after PIVH absorption. This agrees with the literature that points to peripheral and central auditory changes, also affecting the development of auditory skills, whose acquisition directly interferes with language development, school learning, and consequent social performance^9^^,^^15^, ^16^, ^17^.

The presence of potentials at the cortical level signals the arrival of sound information to the auditory cortex, allowing the objective evaluation of the cortical structures involved in attention, localization, memory, and discrimination of sounds, and making it possible to relate their findings to linguistic performance.

The authors of a previous study suggested that such potentials could be used during hospitalization to identify early possible central changes throughout the maturation of preterm NBs^18^. This statement is in line with the objectives of the present research.

The analysis of ABR results found absolute latencies and interpeak intervals within normal parameters in all infants in the CG, in the three evaluations. This demonstrates constant and gradual auditory pathway maturation. However, the analysis of ABR parameters in the SG verified changes in 13 % of the infants, who were referred for monitoring of auditory pathway maturation throughout their development, until around 3-years-old.

A previous study verified central changes in 33.4 % of premature NBs; children with PIVH grades II, III, and IV had a higher occurrence of central changes than those with grade I. In the audiological monitoring at 6-months old, the central auditory change remained in 51.85 % of the children who already had it at birth^7^. However, these findings did not corroborate those obtained in the present study, probably due to the difference in PIVH grades of the study population.

The comparison of potentials did not show any difference between the ears, corroborating other studies whose results also indicated no difference between the ears^5^^,^^19^. However, this disagrees with studies that concluded that auditory function is asymmetrical, with a slight advantage for the right ear^20^^,^^21^.

In the comparative analysis of the mean ABR absolute wave latencies and interpeak intervals between infants in the SG and CG (Tables 1 to 3), the parameters that most differentiated the two groups always had shorter latency times in the EG, in the three periods evaluated. Some authors attribute this shortening to changes in the morphological and functional development of the auditory system, which may characterize transient or permanent neural dysfunctions^22^.

The difference in the interpeak interval III‒V between SG and CG continued at 6-months, now at the expense of wave V, which was also significantly shorter in SG (Table 4). The hypothesis for this finding is that it probably shows accelerated maturation due to a rapid increase in axonal myelin density in the brainstem^24^, expressed by the shorter latency of wave V and/or shorter nerve conduction time due to the shorter length of the nerve fibers of the auditory pathway in the brainstem, in its most rostral region, in infants who had PIVH^23^^,^^24^.

This study aimed to investigate the presence of exogenous CAEP components in preterm NBs. The clinical applications of CAEP include the monitoring of auditory maturation in NBs, since research into the presence or absence of its components allows us to observe the response to sounds from the auditory pathway to the cerebral cortex. When performed longitudinally, it makes it possible to infer how the auditory system is organized in relation to sound reception at the cortical level over time^25^.

The CAEP P1 component has been established as a biological marker to assess the central auditory system maturation in children, as this robust positive response is easily identified and occurs between 100‒300 ms post-stimulus, depending on the child's age^26^.

CAEPs measure cognitive development, especially in premature babies. From birth, their capture shows the organization of cortical generators and the development of the central auditory system^26^^,^^27^. The authors of a study found that children with abnormal CAEPs during the neonatal period may have language alterations^18^.

This study named the CAEP peaks and valleys as P1, N1, P2, N2, despite having late latency times. This agrees with other studies that maintained the same nomenclature, as they attributed such an increase to the immaturity of the cortex structures^17^^,^^25^.

The mean P1, N1, and N2 values (Table 4) were slightly shorter than those found in previous studies – whose results were, respectively: P1 – 247.93 in the right ear and 251.23 in the left ear; N1 – 395.39 in the right ear and 403.77 in the left ear;[25] P1 – 205.0 in the right ear and 201.8 in the left ear; N1 – 298.2 in the right ear and 302.8 in the left ear^17^.

In the first evaluation of this study, the absence of P1 and N1 (Table 2) was higher in both groups than in a previous study^25^. It found absent P1 and N1 in 13 % (*n* = 4) of the neonates in the preterm group, perhaps because they were a group with a higher GA (mean of 34-weeks), fewer complications, and/or shorter length of hospital stay. As for oscillations in the percentage of presence/absence of CAEP in the second and third evaluations, the literature consulted lacked results for comparison. However, both the SG and the CG maintained the absence of these parameters, which may indicate that prematurity is the main risk factor for changes in auditory processing at the cortical level and may be an indicator of cognitive changes or immature cortical structures in this population^26^^,^^27^.

The evolutionary study of the mean ABR and CAEP latencies (Table 5, Table 6; Fig. 3) in SG and CG showed that the ABR waves matured rapidly throughout the study period, mainly in the first 3-months after hospital discharge. This characterizes a phenomenon common to premature infants with accelerated maturation, known as the recovery period or catch-up, already observed in a previous study^19^.

However, wave I matured gradually and continuously, without significant differences between periods. The literature indicates that wave I is already practically mature at birth, even in preterm infants, as it reflects nerve conduction proximal to the cochlea^28^^,^^29^.

The CAEP did not show significant differences throughout the study period, possibly due to their late maturation, beginning at birth, and completing around 14-years old^23^^,^^30^, ^31^, ^32^.

It is interesting to note that the ABR parameters, especially wave V and the I‒V interpeak interval, remained shorter in SG than in CG in the three evaluations. This may indicate accelerated maturation due to a rapid increase in the myelin layer in the auditory nerve in the brainstem region, or synaptic plasticity influenced by the auditory experience (even in a NICU), capable of proliferating collateral axons and dendritic germination and changing the neural response properties^24^^,^^28^.

Fig. 3 shows the maturation process of the ABR and CAEP waves in the two study groups, which developed quite similarly. Thus, it is believed that SG and CG infants would complete the maturation process at similar times. This belief could only be confirmed by monitoring these children’s maturation process until around 3-years old to ensure adequate auditory development and language acquisition and development.

The etiology of prematurity is multifactorial, contributing to the possibility of numerous complications and serious consequences.

One of the limitations of this study was that its sample had mostly grade I PIVH; therefore, the PIVH/PVL duration and severity parameters in SG were not considered, making it impossible to correlate the degree of involvement and the results.

Another aspect that may have interfered with the analysis of the results in this study was that, because they remained in the NICU, some premature infants could only be evaluated a few weeks after their birth, having already experienced several complications during their hospitalization. These complications were not considered. Furthermore, the PIVH may have already been completely resolved by hospital discharge, which leads us to infer that the brain structures previously compressed by the hemorrhage were no longer compressed due to blood absorption, ceasing to be a preponderant factor.

Thus, prematurity is a primary factor for lifelong damage, demonstrating the importance of looking at preterm children with extreme attention from an audiological standpoint, aiming at the full development of their oral and written language, as they have a higher occurrence of minimal neurological dysfunctions, such as attention deficit, hyperactivity, and school performance below expectations^8^^,^^33^.

## Conclusions

This study found a gradual decrease in nerve conduction time towards the most central structures of the auditory pathway in the brainstem in both study groups, corroborating that neuromaturation of the auditory system occurs in the caudo-rostral direction. The comparison of brainstem and cortical potentials showed that auditory function is symmetrical in the peripheral and central portions of the auditory pathway in both groups.

The present study concluded that the maturation of the ABR and CAEP waves in both groups developed in a very similar way over the six months after hospital discharge. Therefore, it is believed that hearing monitoring is necessary until around 3-years of age to ensure adequate auditory and language development.

Prematurity is a primary factor for lifelong damage, demonstrating the importance of looking at preterm children with extreme attention from an audiological standpoint.

## Statements

The Following manuscript has been written in accordance with the CONSORT rules.

In accordance with editorial policy, the authors declare that data relating to the article entitled “Electrophysiological evaluation of the auditory pathway in newborns and infants with peri‑intraventricular hemorrhage and/or periventricular leukomalacia”, should be requested from the corresponding author.

## Authors’ contributions

Rosanna Giaffredo Angrisani: Conceptualization; methodology; validation; formal analysis; data curation; investigation; writing-review & editing; writing-original draft. carla gentile matas: conceptualization; validation; supervision; writing-review & editing.

Natalia Oliveira Balzarini: Methodology; formal analysis; data curation; investigation.

Carla Regina Tragante: Methodology; validation; formal analysis.

Valdenise Martins Laurindo Tuma Calil: Writing-review & editing.

Werther Brunow de Carvalho: Writing-review & editing.

## Data availability statement

The datasets generated and/or analyzed during the current study are available from the corresponding author upon reasonable request.

## Declaration of competing interest

The authors declare no conflicts of interest.
